# Two New Polyketides From *Streptomyces* sp. SA61: Isolation, Structure Elucidation, and Insecticidal Activity Against *Bemisia tabaci*

**DOI:** 10.1002/cbdv.71706

**Published:** 2026-09-15

**Authors:** Pengfei Cui, Jiajun Chen, Zhaoyu Zhu, Fei Liu, Zhiguo Yu

**Affiliations:** ^1^ College of Plant Protection Shenyang Agricultural University Shenyang China

**Keywords:** *Bemisia tabaci*, insecticidal activity, secondary metabolites, *Streptomyces*, structural identification

## Abstract

To discover novel green bio‐based insecticidal lead compounds, six polyketides, including two new compounds, strekingmycin I (**5**) and strekingmycin J (**6**), together with four known analogues, were isolated and identified from the fermentation products of earthworm gut‐derived *Streptomyces* sp. SA61. Their structures were established by comprehensive spectroscopic analysis, including NMR, HR‐ESI‐MS, and single‐crystal x‐ray diffraction. Notably, the crystallographic data of strekingmycin G (**3**) were obtained for the first time, which unambiguously confirmed its absolute configuration. In insecticidal activity assays against the third‐instar nymphs of *Bemisia tabaci*, all six compounds exhibited varying degrees of toxicity. Among them, strekingmycin A (**1**) displayed the most potent activity, with an LC_50_ value of 5.58 µg/mL, which was within the same concentration range as that of the positive control spirotetramat, albeit less active than the commercial standard. This study enriches the structural diversity of *strekingmycin*‐type polyketides by reporting two new derivatives and providing the first crystallographic evidence for compound **3**. Furthermore, it demonstrates for the first time the insecticidal potential of the strekingmycin family against *B. tabaci*, offering a scientific basis for further research on the management of piercing‐sucking pests.

## Graphical abstract

Isolation of six polyketides (including two new compounds, strekingmycins I and J) from earthworm gut‐derived *Streptomyces* sp. SA61 and their insecticidal activity against third‐instar nymphs of *Bemisia tabaci*. Strekingmycin A (**1**) exhibited the most potent activity (LC_50_ = 5.58 µg/mL), comparable to the commercial insecticide spirotetramat.

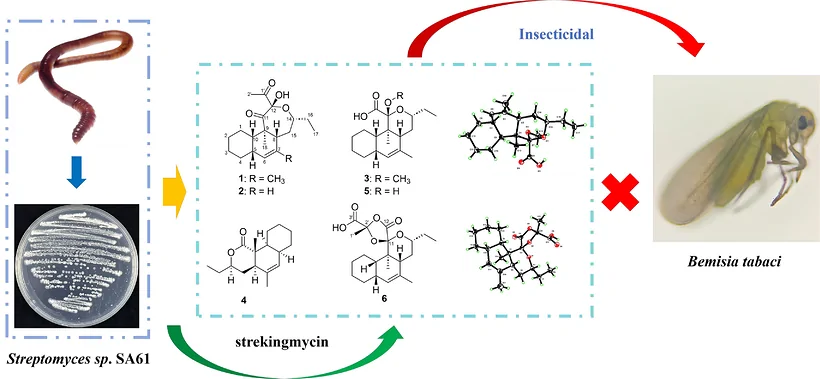

## 1 Introduction

*Bemisia tabaci*, also known as cotton whitefly or sweet potato whitefly, is a species belonging to the order Hemiptera, representing a species complex undergoing rapid evolutionary divergence [1]. It has an extremely wide host range, infesting not only commercial crops such as cucumber, ornamental flowers, and eggplant, but also various weed species. *B. tabaci* exhibits distinct host preferences, with a strong affinity for tomato, cucumber, and sugar beet, while avoiding plants including celery and bitter melon [2, 3]. After feeding on virus‐infected plants, *B. tabaci* can carry and transmit plant viruses to healthy host plants during subsequent feeding, triggering large‐scale viral epidemics in agricultural systems [4, 5, 6, 7]. To date, more than 400 plant viruses, including tomato yellow leaf curl virus, have been documented to be transmissible by *B. tabaci* via its feeding activity [8]. At present, the control of *B. tabaci* mainly relies on chemical insecticides such as neonicotinoids. However, long‐term and excessive application has led to the development of insecticide resistance in field populations of *B. tabaci* against commonly used pesticides [9, 10]. Therefore, the discovery and development of novel control agents are of great significance for the effective and sustainable management of *B. tabaci* [11].

Microbial secondary metabolites are characterized by remarkable structural diversity, specific biological activities, and strain‐dependent biosynthesis. Their chemical scaffolds encompass a wide variety of types, including polyketides, nonribosomal peptides, terpenoids, and alkaloids. Such complex chemical structures endow these metabolites with abundant and unique biological activities [12, 13, 14, 15]. Microbial secondary metabolites have long become a vital resource reservoir for human development. In the medical field, numerous blockbuster clinical drugs, such as antibiotics, antitumor agents, immunosuppressants, and lipid‐lowering medicines, are derived from microbial sources, providing core materials for tackling intractable medical problems including infectious diseases, malignant tumors, and immune disorders [16]. In the agricultural sector, green agrochemicals including biopesticides, plant growth regulators, and biofertilizers achieve the goals of high efficiency, low toxicity, and environmental friendliness based on secondary metabolites, supporting the sustainable development of agriculture [17]. In the industrial domain, their applications in food additives, biomaterials, and environmental remediation are continuously expanding, revealing enormous industrial potential [18, 19, 20].

In this study, *Streptomyces* sp. SA61 was selected as the research subject, and a systematic investigation into the isolation and structural identification of its secondary metabolites was undertaken. The insecticidal activity of the obtained compounds against *B. tabaci* was evaluated via stomach toxicity assays, and all compounds displayed moderate insecticidal activity. This study enriches the structural diversity of secondary metabolites derived from *Streptomyces* and provides a research basis for the development of novel bio‐based insecticides.

## 2 Results and Discussion

### 2.1 Structural Identification of Known Compounds **1–4**

By comparing the experimental spectral data with those reported in the literature [21, 22, 23], compounds **1**–**4** were identified as known polyketides (Figure 1): strekingmycin A (**1**), strekingmycin B (**2**), strekingmycin G (**3**), and PI‐200 (**4**). The detailed NMR data are provided in (Figures S1–S8).

**FIGURE 1:**
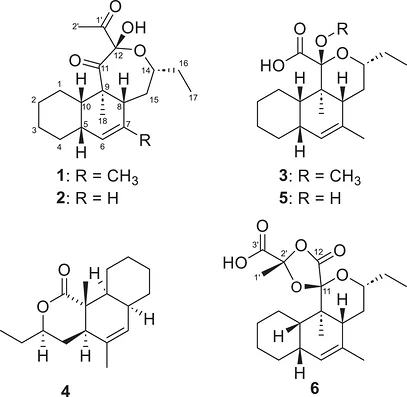
Chemical Structures of Compounds **1–6**.

### 2.2 Structural Identification of New Compounds **5** and **6**

Compound **5** was obtained as a white powder, readily soluble in methanol. Its molecular formula was determined to be C_18_H_28_O_4_ with 5 degrees of unsaturation, based on the HR‐ESI‐MS ion peak at *m/z* obsd 309.2072 [M+H]^+^ (Figure S15) and ^13^C NMR spectral data (Table 1).

**TABLE 1:** ^1^H NMR (600 MHz) and ^13^C NMR (150 MHz) data of Compounds **5** and **6** (in CDCl_3_).

NO	5		6	
	*δ*_H_ (*J* in Hz)	*δ*_C_	*δ*_H_ (*J* in Hz)	*δ*_C_
1	1.37 (m, 1H)	27.4	1.48 (m, 1H)	26.1
	0.93 (m, 1H)		1.37 (m, 1H)	
2	1.58 (m, 1H)	25.7	1.48 (m, 1H)	22.4
	1.22 (m, 1H)		1.37 (m, 1H)	
3	1.47 (m, 1H)	23.1	1.65 (m, 1H)	24.7
	1.31 (m, 1H)		1.37 (m, 1H)	
4	1.73 (m, 1H)	29.7	1.48 (m, 2H)	28.0
	1.65 (m, 1H)			
5	2.28 (s, 1H)	35.6	2.18 (s, 1H)	36.4
6	5.50 (d, *J* = 3.1 Hz, 1H)	127.8	5.36 (s, 1H)	127.4
7		135.7		131.9
8	2.82 (t, *J* = 8.6 Hz, 1H)	38.9	2.64 (dd, *J* = 13.4, 3.7 Hz, 1H)	41.7
9		44.8		41.9
10	2.30 (dd, *J* = 10.8, 5.7 Hz, 1H)	38.7	2.05 (q, *J* = 6.4 Hz, 1H)	37.4
11		99.4		101.9
12		172.7		167.3
14	3.98 (m, 1H)	73.8	3.86 (dd, *J* = 7.5, 4.4 Hz, 1H)	74.4
15	1.65 (m, 2H)	28.5	1.65 (m, 1H)	28.7
			1.48 (m, 1H)	
16	1.65 (m, 1H)	28.9	1.48 (m, 2H)	28.6
	1.60 (m, 1H)			
17	0.97 (t, *J* = 7.4 Hz, 3H)	9.9	0.84 (t, *J* = 7.4 Hz, 3H)	9.5
18	0.90 (s, 3H)	14.5	1.28 (s, 3H)	13.3
19	1.68 (s, 3H)	19.6	1.62 (s, 3H)	20.6
1’			1.79 (s, 3H)	22.9
2’				102.2
3’				173.1

The ^1^H NMR spectrum (Figure S9) showed one olefinic proton at *δ*_H_ 5.50 (d, *J* = 3.1 Hz, 1H, H‑6), one oxygen‐bearing methine proton at *δ*_H_ 3.98 (m, 1H, H‐14), and three methyl protons at *δ*_H_ 1.68 (s, 3H, H‐19), 0.97 (t, *J* = 7.4 Hz, 3H, H‐17), and 0.90 (s, 3H, H‐18). The ^13^C NMR (Figure S10) and HSQC (Figure S12) spectra revealed 18 carbon signals, including one carbonyl carbon (C‐12, *δ*_C_ 172.7), two olefinic carbons (C‐7,*δ*_C_ 135.7; C‐6, *δ*_C_ 127.8), one hemiketal carbon (C‐11, *δ*_C_ 99.4), one oxygen‐bearing methine carbon (C‐14, *δ*_C_ 73.8), one quaternary carbon (C‐9, *δ*_C_ 44.8), three methyl carbons (C‐19, *δ*_C_ 19.6; C‐18, *δ*_C_ 14.5; C‐17, *δ*_C_ 9.9), and nine upfield aliphatic carbons. Since the above structural units only accounted for 2 degrees of unsaturation, combined with the 5 degrees of unsaturation derived from the molecular formula, compound **5** was deduced to possess a tricyclic fused skeleton and belong to strekingmycin‐type polyketides.

The planar structure was further confirmed by the key correlations observed in the HMBC spectrum (Figure S13). H‐18 showed correlations with C‐8, C‐9, C‐10, and C‐11. Combined with the chemical shift of C‐11 (*δ*_C_ 99.4), it was confirmed that C‐9 was substituted by a methyl group and C‐11 was substituted by a hydroxy group. H‐19 exhibited correlations with C‐6, C‐7, and C‐8, and H‐6 showed correlations with C‐8 and C‐19, indicating the presence of a double bond between C‐6 and C‐7 with a methyl group attached to C‐7. H‐17 correlated with C‐14 and C‐16; combined with the triplet coupling characteristic of H‐17, it was determined that C‐14 was connected to an ethyl group. Meanwhile, the ^1^H‐^1^H COSY spectrum (Figure S11) revealed two fatty chain spin systems: H‐6/H‐5/H‐4/H‐3/H‐2/H‐1/H‐10/H‐5 and H‐8/H‐15/H‐14/H‐16/H‐17, which further comprehensively supported the planar structure.

**FIGURE 2:**
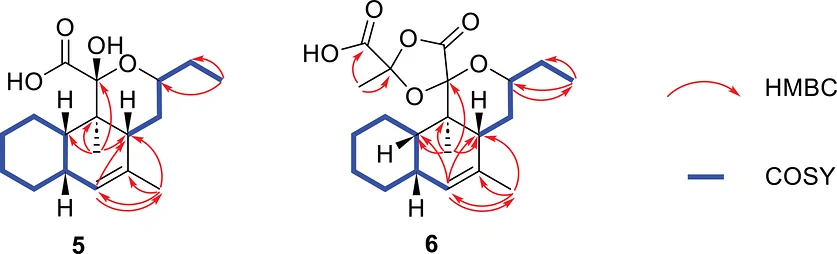
X‐ray crystallographic structure of compounds **3** (left) and **6** (right).

**FIGURE 3:**
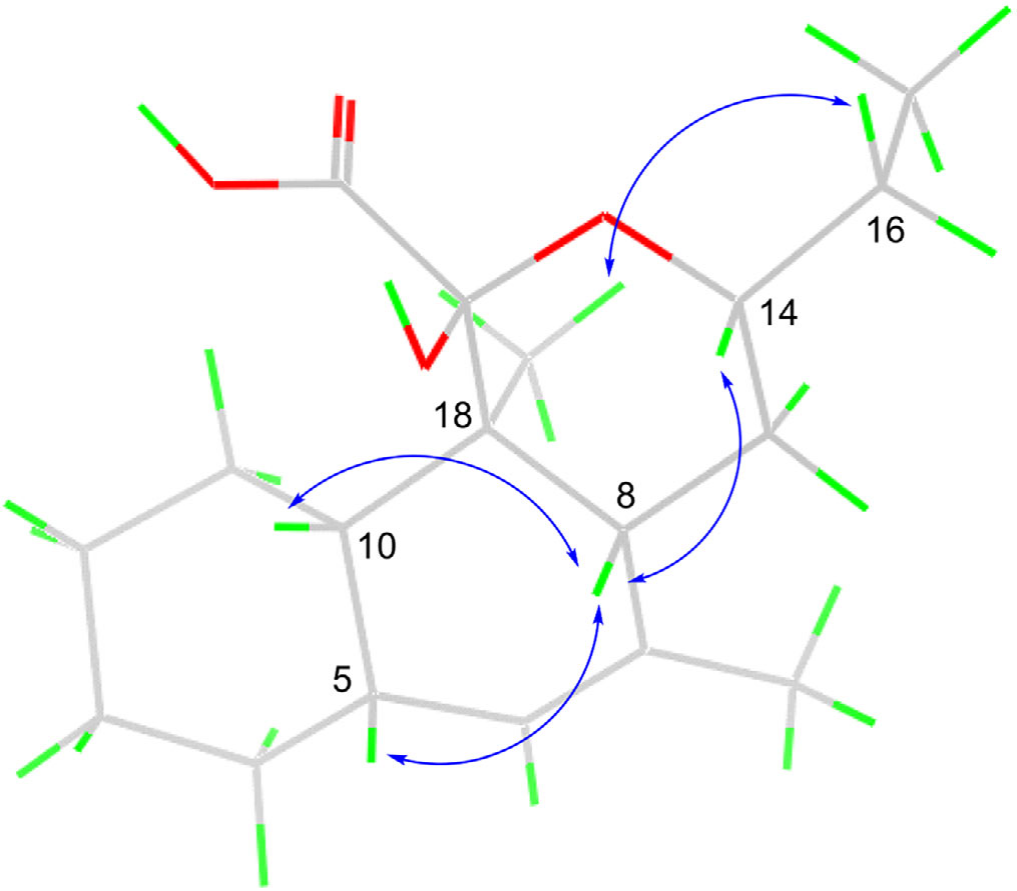
Key NOESY correlations of compound **5**.

In the present study, the absolute configuration of compound **3** was unambiguously determined by single‐crystal x‐ray diffraction (Figure 2), providing a solid reference for assigning the configuration of compound **5**. In the NOESY spectrum (Figure S14), the correlations of H‐5/H‐8, H‐8/H‐10, and H‐8/H‐14 indicated that these protons were cofacial in the *β*‐orientation. The correlation between H‐18 and H‐16 suggested that they were positioned on the opposite face in the *α*‐orientation. By analogy with compound **3**, the hydroxy group at C‐11 was determined to be *β*‐oriented (Figure 3). Based on the above evidence, compound **5** was identified and named strekingmycin I.

Compound **6** was obtained as a white powder, readily soluble in methanol. Its molecular formula was determined to be C_21_H_30_O_6_ with 7 degrees of unsaturation, based on the HR‐ESI‐MS ion peak at *m/z* obsd 401.1944 [M+Na]^+^ (Figure S22) and ^13^C NMR spectral data (Table 1).

According to the ^1^H NMR spectral data (Figure S16), the compound displayed one olefinic proton signal at *δ*_H_ 5.36 (s, 1H, H‐6), one oxygen‐bearing methine proton at *δ*_H_ 3.86 (dd, *J* = 7.5, 4.4 Hz, 1H, H‐14), and four methyl proton signals at *δ*_H_ 1.79 (s, 3H, H‐1′), 1.62 (s, 3H, H‐19), 1.28 (s, 3H, H‐18), and 0.84 (t, *J* = 7.4 Hz, 3H, H‐17). Combined with the ^13^C NMR (Figure S17) and HSQC spectra (Figure S19), the compound showed 21 carbon signals in total, including two carbonyl carbons at *δ*_C_ 173.1 (C‐3′) and *δ*_C_ 167.3 (C‐12), two olefinic carbons at *δ*_C_ 131.9 (C‐7) and *δ*_C_ 127.4 (C‐6), two hemiketal carbons at *δ*_C_ 102.2 (C‐2’) and *δ*_C_ 101.9 (C‐11), one oxygen‐bearing methine carbon (C‐14) at *δ*_C_ 74.4, one quaternary carbon (C‐9) at *δ*_C_ 41.9, four methyl carbons at *δ*_C_ 22.9 (C‐1’), *δ*_C_ 20.6 (C‐19), *δ*_C_ 13.3 (C‐18), and *δ*_C_ 9.5 (C‐17), as well as nine upfield aliphatic carbons. The above structural units only contributed 3 degrees of unsaturation. Combined with the 7 degrees of unsaturation derived from the molecular formula, the molecule was established to possess a tetracyclic fused skeleton and belong to strekingmycin‐type tetracyclic polyketide derivatives.

**FIGURE 4:**
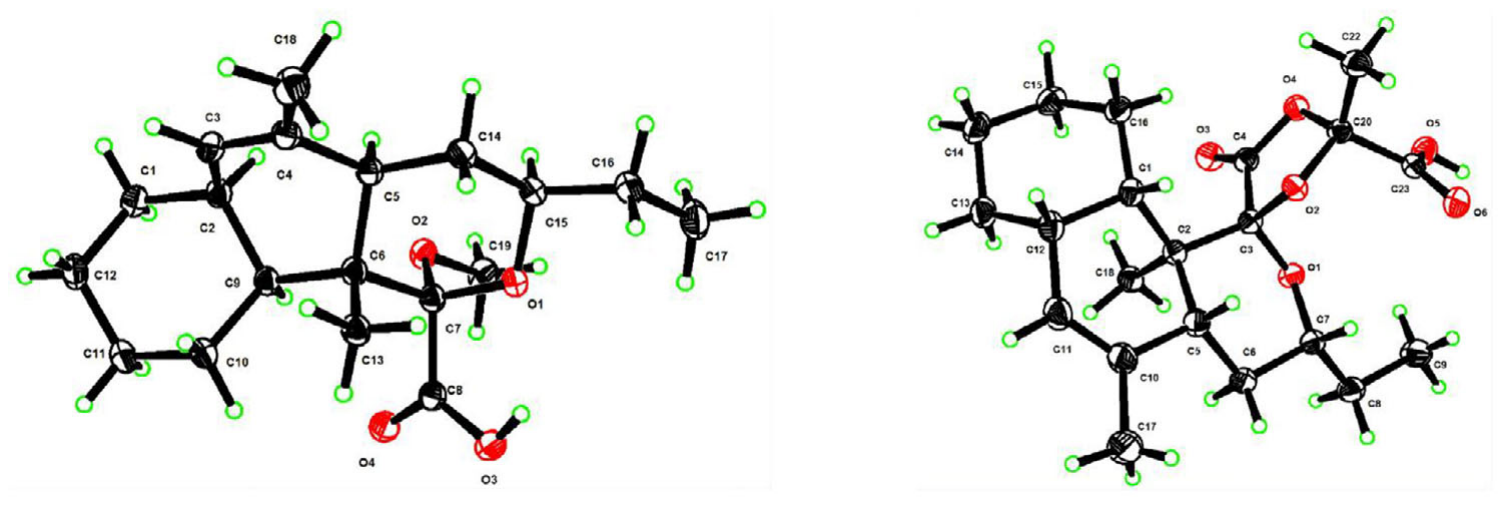
Key ^1^H‐^1^H COSY and significant HMBC correlation of compounds **5** and **6**.

The planar structure was further confirmed by the HMBC (Figure S20) and ^1^H‐^1^H COSY correlations (Figure S18). H‐18 showed correlations with C‐8, C‐9, C‐10, and C‐11. Combined with the chemical shift of C‐11 (*δ*_C_ 101.9), it was confirmed that C‐9 was substituted by a methyl group and C‐11 existed as a hemiketal moiety. H‐19 exhibited correlations with C‐6, C‐7, and C‐8, and H‐6 showed correlations with C‐8 and C‐19, indicating the presence of a double bond between C‐6 and C‐7 with a methyl group attached to C‐7. H‐17 correlated with C‐14 and C‐16; combined with the triplet coupling characteristic of H‐17, it was determined that C‐14 was connected to an ethyl group. Meanwhile, the ^1^H‐^1^H COSY spectrum revealed two fatty chain spin systems: H‐6/H‐5/H‐4/H‐3/H‐2/H‐1/H‐10/H‐5 and H‐8/H‐15/H‐14/H‐16/H‐17, which fully established the tetracyclic skeleton of the molecule (Figure 4). Systematic comparison of the NMR data of this compound with those of the known compound strekingmycin E [21] showed that they shared an identical tetracyclic skeleton, with the only key difference being the substituent at C‐2’: strekingmycin E had a methyl ester group at C‐2’, whereas this compound contained a free carboxyl group at C‐2’. Their polarity and spectral signals differed significantly, which further validated the accuracy and reliability of the structural elucidation.

The absolute configuration of this compound was ultimately determined by single‐crystal x‐ray diffraction (Figure 2). The crystal data have been deposited at the Cambridge Crystallographic Data Centre (CCDC) with the deposition number of 2536754. The stereochemical assignment was unequivocal. Combined with the ^2^D NMR correlations (Figures S18–S21)and comparison with its congeners, the structure of compound **6** was fully elucidated and it was consequently named strekingmycin J.

### 2.3 Comparison With Known Strekingmycins

Compound **5** shares a highly similar tricyclic ketal skeleton with the known compound strekingmycin G (**3**). The key structural distinction lies at C‐11: compound 3 bears a methoxy group (*δ*_C_ 104.4), whereas compound **5** contains a hydroxy group (*δ*_C_ 99.4). This difference results in altered hydrogen‐bonding capacity and polarity between the two analogues.

Compound **6** possesses the characteristic tetracyclic skeleton of strekingmycin‐type polyketides. Compared with the known compound strekingmycin E [21], the distinctive feature of **6** is the presence of a free carboxyl group at C‐2’ instead of the methyl ester group, which also modifies the molecular polarity and hydrogen‐bonding potential.

In addition, although compound **3** has been reported previously [22], its absolute configuration was inferred solely from NMR data. In the present study, single crystals of **3** were successfully obtained for the first time, and its absolute configuration was unambiguously established by single‐crystal x‐ray diffraction (CCDC 2541963; Figure 2), thereby filling the crystallographic data gap for this compound.

### 2.4 Insecticidal Activity Assay

The insecticidal activity assay results (Table 2) revealed that all six compounds exhibited varying degrees of toxicity against *B. tabaci*, indicating that the strekingmycin‐type polyketide skeleton possesses moderate insecticidal potential. At a concentration of 100 µg/mL after 48 h of treatment, the corrected mortality rates of compounds **1–6** against *B. tabaci* were 91.36%, 75.31%, 53.66%, 42.17%, 69.51%, and 76.54%, respectively. Among them, compound **1** (strekingmycin A) displayed the most potent activity, with an LC_50_ value of 5.58 µg/mL. This compound exhibited toxicity within the same concentration range as the positive control spirotetramat (LC_50_ = 2.29 µg/mL), albeit approximately 2.4‐fold less active than the commercial standard.

**TABLE 2:** Insecticidal activity of compounds **1–6** and spirotetramat against *Bemisia tabaci* at 48 h.

Compounds	Toxicity regression equation (*y*)	^a^LC_50_ (95% CL) (µg/mL)	^b^ *R* ^2^	*p* values
Strekingmycin A (**1**)	*y* = −0.59 + 0.83 *x*	5.58 ± 0.84 (4.48 ‐ 8.40)	0.976	0.854
Strekingmycin B (**2**)	*y* = −0.74 + 0.82 *x*	9.61 ± 1.32 (7.03 ‐ 13.14)	0.968	0.745
Strekingmycin G (**3**)	*y* = −1.49 + 0.80 *x*	72.98 ± 8.63 (56.12 ‐ 94.93)	0.957	0.411
PI ‐ 200 (**4**)	*y* = −1.62 + 1.79 *x*	112.02 ± 15.87 (81.91‐162.92)	0.962	0.996
Strekingmycin I (**5**)	*y* = −1.35 + 1.99 *x*	23.57 ± 3.12 (17.42 ‐ 31.93)	0.941	0.570
Strekingmycin J (**6**)	*y* = −2.43 + 1.79 *x*	37.75 ± 4.28 (29.31 ‐ 48.62)	0.933	0.898
Spirotetramat	*y =* −0.72 + 2.04 *x*	2.29 ± 0.17 (1.94 ‐ 2.67)	0.989	0.962

^a^ LC_50_, 50% lethal concentration; CL, confidence limit

^b^ *R* ^2^, Coefficient of determination, reflecting the goodness‐of‐fit of the dose‐response regression model. Data are the average of three replicates.

Combined with our previous findings on the insecticidal activity of these compounds against *Myzus persicae* (Hemiptera: Aphididae) and *Trialeurodes vaporariorum* (Hemiptera: Aleyrodidae) [21, 22], strekingmycin‐type polyketides exhibit notable toxicity against multiple piercing‐sucking pests, suggesting their potential for further exploration in the context of pest management.

Although the limited number of compounds obtained in this study precludes definitive conclusions regarding structure–activity relationships (SAR), several preliminary observations merit attention in future analogue design. Compound **1** (bearing a carbonyl group at C‐11) exhibited significantly higher activity than compounds **3** (C‐11 methoxy) and 5 (C‐11 hydroxy), suggesting that the substituent at C‐11 may play a critical role in target interaction. Furthermore, the tricyclic compound **1** displayed greater activity than the tetracyclic compound **6**, implying that ring system complexity may influence bioactivity; however, this hypothesis requires validation through the evaluation of additional analogues with controlled structural variations. These preliminary findings provide valuable clues for future structural optimization and mechanistic studies.

### 2.5 Limitations and Future Directions

Several limitations of the present study should be acknowledged. First, the insecticidal activity was evaluated exclusively against third‐instar nymphs of *B. tabaci* under laboratory conditions; field efficacy, residual activity, and activity against other life stages (e.g., adults or eggs) remain to be determined. Second, the mode of action and molecular target of the strekingmycin‐type polyketides have not yet been investigated. The observed insecticidal activity may arise from interference with insect growth, nervous system function, or other physiological processes, and further biochemical and genetic studies are required for target identification. Third, owing to the limited number of compounds obtained, the SAR observations discussed above are preliminary and should be validated through systematic structural modifications and the testing of additional analogues.

Future work will focus on: (i) elucidation of the biosynthetic gene cluster of strekingmycins to delineate their biosynthetic pathway; (ii) transcriptomic or proteomic profiling to investigate the mode of action and identify potential molecular targets; and (iii) field efficacy evaluation and formulation development to facilitate practical agricultural application.

## 3 Conclusions

In this study, six polyketides were systematically isolated and identified from the fermentation products of earthworm gut‐derived *Streptomyces* sp. SA61, comprising two new compounds, strekingmycin I (**5**) and strekingmycin J (**6**), along with four known compounds, strekingmycin A (**1**), strekingmycin B (**2**), strekingmycin G (**3**) and PI‐200 (**4**). The planar structures and absolute configurations of all compounds were established by NMR, HR‐ESI‐MS, and single‐crystal x‐ray diffraction, with the crystallographic data of strekingmycin G (**3**) being obtained for the first time.

Insecticidal activity assays revealed that all six compounds exhibited varying degrees of toxicity against the third‐instar nymphs of *B. tabaci*, with strekingmycin A (**1**) displaying the most potent activity. Its toxicity fell within the same concentration range as that of the commercial insecticide spirotetramat, albeit weaker than the positive control, indicating that **1** possesses promising potential as an insecticidal lead compound.

This study not only enriches the structural diversity of polyketide secondary metabolites from *Streptomyces*, but also differs substantially from our previous report on *T. vaporariorum* [22] in the following respects: (i) it targets *B. tabaci*, an economically important pest with distinct susceptibility profiles; (ii) it identifies two new compounds (**5** and **6**) with novel substitution patterns; and (iii) it provides, for the first time, crystallographic evidence confirming the absolute configuration of strekingmycin G (**3**). Collectively, these findings offer new chemical entities and establish a scientific foundation for the development of novel bio‐based insecticides.

## 4 Experimental Section

### 4.1 General Experimental Procedures

NMR spectra were recorded at room temperature on a Bruker Avance‐600 spectrometer (Bruker BioSpin GmbH, Karlsruhe, Germany). HR‐ESI‐MS data were acquired using a Waters Xevo G2‐XS Q‐TOF mass spectrometer (Waters Corporation, Milford, MA, USA). Column chromatography was performed on 100–200 and 200–300 mesh silica gel (Qingdao Haiyang Chemical Co., Ltd., Qingdao, China), and Sephadex LH‐20 (GE Healthcare, Uppsala, Sweden) was used for gel filtration. Analytical HPLC was carried out on an Agilent 1260 system equipped with a ZORBAX Eclipse XDB‐C18 column (250 × 4.6 mm, 5 µm; Agilent Technologies, Santa Clara, CA, USA), while semi‐preparative HPLC was performed on the same system using a ZORBAX Eclipse XDB‐C18 column (250 × 9.4 mm, 5 µm). All other chemical reagents used in this study were purchased from Tianjin Fuyu Fine Chemical Co., Ltd. (Tianjin, China).

### 4.2 Test Strain and Test Insect

*Streptomyces* sp. SA61 was isolated from the gut of earthworms collected in the laboratory of Microbial Secondary Metabolites, College of Plant Protection, Shenyang Agricultural University, Shenyang, Liaoning Province, China.

The initial population of *B. tabaci* used in this study was obtained from the Insect‐Microbe Symbiosis Laboratory, College of Plant Protection, Shenyang Agricultural University. The insects were reared on *Nicotiana benthamiana* plants in a climate‐controlled chamber under conditions of 25°C ± 2°C, 65%–85% relative humidity, and a photoperiod of L:D = 14:10 h.

### 4.3 Fermentation and Extraction

*Streptomyces* sp. SA61, previously preserved at −80°C, was activated on Gauze's No. 1 medium for 5 days. A single colony was then inoculated into a 20 mL test tube containing 5 mL of sterile ISP2 medium and incubated on a rotary shaker at 180 r/min and 28°C for 48 h to obtain the primary seed culture. The primary seed culture was subsequently transferred into a 250 mL Erlenmeyer flask containing 50 mL of ISP2 medium and incubated under the same conditions for 12 h to generate the secondary seed culture. This secondary culture was then inoculated into 2000 mL Erlenmeyer flasks, each containing 400 mL of liquid F medium (composition per liter: soluble starch 94 g, sucrose 10 g, glucose 10 g, casamino acids 0.1 g, yeast extract 5 g, KNO_3_ 3.3 g, K_2_SO_4_ 0.25 g, MgCl_2_·6H_2_O 10 g), supplemented with 5% (*w*/*v*) macroporous adsorbent resin. Fermentation was carried out at 180 r/min and 28°C for 7 days, with a total fermentation volume of 48 L.

The macroporous resin was collected by filtration, washed with distilled water, and dried at 28°C for 48 h. The dried resin was then eluted by repeated soaking in methanol; the combined extracts were concentrated to dryness under reduced pressure. The resulting residue was partitioned with a mixture of dichloromethane‐methanol‐water (2:1:1, *v/v/v*), and the organic layer was evaporated to afford 32 g of crude extract. This crude material was subjected to silica gel column chromatography eluted with a stepwise gradient of dichloromethane‐methanol (100:0, 100:1, 100:4, 100:8, 100:16, 1:1, 0:100, *v/v*), with 2000 mL of each solvent system. Fractions were monitored by TLC and combined based on similar profiles, ultimately yielding seven fractions designated A‐G.

### 4.4 Isolation and Purification

Fraction A (6.6 g) was subjected to silica gel column chromatography with gradient elution using ***V***_petroleum ether_: ***V***_ethyl acetate_ = 100:1, 100:2, 100:4, 100:8, 100:16, 1:1, 0:100. Similar fractions were detected and combined by TLC to afford three subfractions (AA, AB, and AC). Subfraction AA was further purified by Sephadex LH‐20 column chromatography eluted with ***V***_petroleum ether_: ***V***_dichloromethane_: ***V***_methanol_ = 2:1:1, and identical fractions were pooled by TLC monitoring. Further purification was performed by high‐performance liquid chromatography (HPLC) under the following conditions: ***V***_methanol_: ***V***_water_ = 85:15, 25°C, flow rate 3 mL/min, detection wavelength 210 nm, and *t*R = 22.86 min, yielding compound **1** (1 g).

Subfraction AB was subjected to Sephadex LH‐20 column chromatography eluted with ***V***_dichloromethane_: ***V***_methanol_ = 1:1. Similar fractions were monitored and combined by TLC, then further purified by preparative HPLC under the following conditions: ***V***_methanol_: ***V***_water_ = 80:20, 25°C, flow rate 3 mL/min, detection wavelength 210 nm, and *t*R = 20.76 min, yielding compound **4** (5 mg).

Subfraction AC was subjected to Sephadex LH‐20 column chromatography eluted with ***V***_petroleum ether_: ***V***_dichloromethane_: ***V***_methanol_ = 2:1:1. Similar fractions were monitored and combined by TLC, then further purified by preparative HPLC under the following conditions: ***V***_methanol_: ***V***_water_ = 85:15, 25°C, flow rate 3 mL/min, detection wavelength 210 nm, and *t*R = 26.52 min, yielding compound **2** (6.1 mg).

Fraction C (1.42 g) was subjected to silica gel column chromatography with gradient elution using ***V***_petroleum ether (0.1% HCOOH)_: ***V***_ethyl acetate (0.1% HCOOH)_ = 10:1, 9:1, 8:2, 7:3, 6:4, 5:5, 0:10. The main fractions were monitored and combined by TLC. Further purification was performed on a Sephadex LH‐20 column eluted with ***V***_petroleum ether_: ***V***_dichloromethane_: ***V***_methanol_ = 2:1:1, and similar fractions were pooled by TLC. Finally, purification by HPLC using ***V***_methanol_: ***V***_water_ = 83:17, 25°C, *t*R = 28.61 min afforded compound **3** (25 mg).

Fraction D (2.16 g) was subjected to silica gel column chromatography with gradient elution using ***V***_petroleum ether_: ***V***_ethyl acetate_ = 10:1, 9:1, 8:2, 7:3, 6:4, 5:5, 0:10. The target fractions were combined according to TLC results and further separated by Sephadex LH‐20 column chromatography eluted with ***V***_dichloromethane_: ***V***_methanol_ = 1:1. Finally, purification by HPLC using ***V***_acetonitrile (0.1% HCOOH)_: ***V***_water (0.1% HCOOH)_ = 60:40, at 25°C, flow rate 3 mL/min, detection wavelength 254 nm, and *t*R = 27.37 min afforded compound **5** (2 mg).

Fraction G (2.76 g) was subjected to silica gel column chromatography with gradient elution using ***V***_petroleum ether_: ***V***_ethyl acetate_ = 10:1, 9:1, 8:2, 7:3, 6:4, 5:5, 0:10. The main fractions were monitored and combined by TLC, then further separated by Sephadex LH‐20 column chromatography eluted with ***V***_petroleum ether_: ***V***_dichloromethane_: ***V***_methanol_ = 2:1:1. Finally, purification by HPLC using ***V***_methanol (0.1% HCOOH)_: ***V***_water (0.1% HCOOH)_ = 82:18, at 25°C, flow rate 3 mL/min, detection wavelength 210 nm, and *t*R = 24.01 min afforded compound **6** (15 mg).

### 4.5 Structural Characterization

Compound **5**: White powder, odorless. The ^1^H NMR and ^13^C NMR data are listed in (Table 1). HR‐ESI‐MS: *m/z* 309.2072 [M+H]^+^ (calcd for [C_18_H_29_O_4_]^+^, 309.2066).

Compound **6**: White powder, odorless. The ^1^H NMR and ^13^C NMR data are listed in (Table 1). HR‐ESI‐MS: *m/z* 401.1944 [M+Na]^+^ (calcd for [C_21_H_30_NaO_6_]^+^, 401.1940).

### 4.6 Crystallographic Data of Compounds **3** and **6**

Compound **3**:C_19_H_30_O_4_, *M* = 322.43, *T* = 170 K, space group P2_1_2_1_2_1_, *a* = 8.2729 (9) Å, *b* = 10.9273 (9) Å, *c* = 19.993 (2) Å, *ɑ* = *γ* = *β* = 90°, *V* = 1807.4 (3) Å^3^, *Z* = 4, *μ* (Cu K*ɑ*) = 0.651 mm^−1^, *Dcalc* = 1.185 g/cm^−3^, *F*(000) = 704.0, A total of 3711 reflections were collected in the range of 0° ≤ 2θ ≤ 149.422°, of which 3695 independent reflections were selected for analysis with *I* > 2σ(*I*). Final *R*_1_ = 0.0390, w*R*_2_ = 0.0984 (all data), goodness of fit = 1.072.

Compound **6**:C_21_H_30_O_6_, *M* = 378.45, *T* = 170 K, space group P2_1_, *a* = 10.0294 (6) Å, *b* = 6.2168 (4) Å, *c* = 15.894 (1) Å, *ɑ* = *γ* = 90°, *β* = 92.956(2)°,*V* = 989.68(11) Å^3^, *Z* = 2, *μ* (Cu K*ɑ*) = 0.754 mm^−1^, *Dcalc* = 1.270 g/cm^−3^, *F*(000) = 408.0, A total of 4059 reflections were collected in the range of 0° ≤ 2θ ≤ 148.942°, of which 3886 independent reflections were selected for analysis with *I* > 2σ(*I*). Final *R*_1_ = 0.0366, w*R*_2_ = 0.0968 (all data), goodness of fit = 1.061.

### 4.7 Insecticidal Activity Assay

The insecticidal activity of compounds **1–6** against *B. tabaci* was evaluated using a modified leaf‐dipping method based on a previously reported protocol [24]. The test insects used in this study were of the **MEAM1** cryptic species, which had been maintained on *Nicotiana benthamiana* plants for multiple generations under controlled laboratory conditions. The population had no history of long‐term insecticide exposure and remained susceptible to conventional insecticides. Rearing conditions were maintained at 25°C ± 2°C, 65%–85% relative humidity, and a photoperiod of 14 h light: 10 h dark(L: D = 14: 10).

To obtain synchronized cohorts of uniform developmental stage, a synchronized oviposition and hatching protocol was employed. Healthy adult whiteflies were introduced onto tobacco leaves for egg deposition over a 24 h period, after which the adults were removed. The eggs were allowed to develop for 7–9 days. Third‐instar nymphs were selected under a stereomicroscope based on the following morphological criteria: body flattened, elliptical in shape, pale yellowish‐green in color, 0.50–0.60 mm in length, with faint reddish eye spots and no prominent red compound eyes [25].

Mature functional leaves free from damage or insect spots were excised from the middle part of 4‐ to 5‐week‐old tobacco plants and trimmed to uniform size for subsequent use. Test compounds were dissolved in 0.3% acetone, supplemented with 0.5% Tween‐80, and then serially diluted with 30% sucrose solution to five concentrations: 100, 50, 25, 12.5, and 6.25 µg/mL. The final acetone concentration was kept consistent across all treatment groups, while the blank control was prepared with sucrose solution containing equivalent amounts of acetone and 0.5% Tween‐80 to eliminate any potential solvent effects on the test insects. Spirotetramat was used as the positive control at a concentration of 6.25 µg/mL. The prepared tobacco leaf discs were immersed in the respective test solutions for 1 min, then removed and air‐dried at room temperature in the dark.

Each well, containing one treated leaf disc, was regarded as a single biological replicate. Thirty healthy third‐instar nymphs, selected as described above, were introduced into each well, and the opening was covered with gauze to prevent escape. Three independent biological replicates were conducted for each compound concentration, giving a total of 90 nymphs tested per concentration. Mortality was recorded 48 h posttreatment. Nymphs were considered dead if no movement, including twisting or stretching of the body, was observed upon gentle probing with a fine brush. The natural mortality rate in the blank control group remained below 10% at 48 h posttreatment.

### 4.8 Statistical Analysis

All insecticidal activity assays were performed in triplicate, and data are expressed as mean ± standard deviation (SD). Concentration–response data were analyzed by probit regression using SPSS Statistics (version 26.0, IBM Corp., Armonk, NY, USA) to calculate the median lethal concentration (LC_50_) and its 95% confidence interval. The goodness‐of‐fit of the regression model was assessed using the coefficient of determination (*R^2^*). Mortality and corrected mortality rates of *B. tabaci* were calculated according to Formulae (1) and (2), respectively. Statistical differences among treatment groups were determined by one‐way analysis of variance (ANOVA) followed by Tukey's post hoc test, with *p* < 0.05 considered statistically significant.

(1) $$M/\%=\frac{N_{d}}{N_{t}}\times 100$$

(2) $$\overset{-}{M}/\%=\frac{M_{t}-M_{c}}{100-M_{c}}\times 100$$

*Note*: In the formulas, *N*_d_ represents the number of dead insects in the treatment group; *N*_t_ represents the total number of insects in the corresponding group; *M*_t_ represents the mortality rate of the treatment group (%); *M*_c_ represents the corrected mortality rate of the treatment group (%).

## Author Contributions

**Zhiguo Yu**: designed the study. **Pengfei Cui**, **Jiajun Chen**, **Zhaoyu Zhu**, and **Fei Liu**: performed the experiments. **Pengfei Cui** and **Fei Liu**: conducted the strain fermentation and structural elucidation. **Pengfei Cui, Jiajun Chen**, and **Zhaoyu Zhu**: carried out the activity assays and data analysis, and drafted the manuscript. All authors contributed to the final revision of the paper.

## Conflicts of Interest

The authors declare no conflicts of interest.

## Supporting information




**Supporting File 1**: cbdv71706‐sup‐0001‐SuppMat.docx.

## Data Availability

The data that supports the findings of this study are available in the Supporting Information of this article.

## References

[1] H. Wang , S. Geng , S. Liu , et al., “Unraveling the Cryptic *Bemisia tabaci* Species Complex: Global Phylogenomic Analysis Reveals Evolutionary Relationships and Biogeographic Patterns,” Insect Science 33 (2026): 418–440, 10.1111/1744-7917.13501. 39930607

[2] M. M. Rahman Shah , Z. Zhang , J. Hu , A. Gaber , and A. Hossain , “Impact of Leaf Trichomes of Tomatoes and Weeds on the Host Selection and Developmental Bioassays of *Bemisia tabaci* Q and A Cryptic Species,” Heliyon 9 (2023): e20077, 10.1016/j.heliyon.2023.e20077. 37809545 PMC10559822

[3] S. Gautam , H. Mugerwa , J. Buck , et al., “Differential Transmission of Old and New World Begomoviruses by Middle East‐Asia Minor 1 (MEAM1) and Mediterranean (MED) Cryptic Species of *Bemisia tabaci* ,” Viruses 14 (2022): 1104, 10.3390/v14051104. 35632844 PMC9146840

[4] W. Islam , K. S. Akutse , M. Qasim , et al., “ *Bemisia tabaci*‐Mediated Facilitation in Diversity of Begomoviruses: Evidence From Recent Molecular Studies,” Microbial Pathogenesis 123 (2018): 162–168, 10.1016/j.micpath.2018.07.008. 30017827

[5] E. Fiallo‐Olivé and J. Navas‐Castillo , “Begomoviruses: What Is the Secret(s) of Their Success?,” Trends in Plant Science 28 (2023): 715–727, 10.1016/j.tplants.2023.01.012. 36805143

[6] Y.‐M. Wang , Y.‐Z. He , X.‐T. Ye , et al., “A Balance Between Vector Survival and Virus Transmission Is Achieved Through JAK/STAT Signaling Inhibition by a Plant Virus,” Proceedings of the National Academy of Sciences 119 (2022): e2122099119, 10.1073/pnas.2122099119. PMC9564230 36191206

[7] B. Liu , E. L. Preisser , D. Chu , et al., “Multiple Forms of Vector Manipulation by a Plant‐Infecting Virus: *Bemisia tabaci* and Tomato Yellow Leaf Curl Virus,” Journal of Virology 87 (2013): 4929–4937, 10.1128/jvi.03571-12. 23408638 PMC3624301

[8] R. L. Gilbertson , O. Batuman , C. G. Webster , and S. Adkins , “Role of the Insect Supervectors *Bemisia tabaci* and Frankliniella Occidentalis in the Emergence and Global Spread of Plant Viruses,” Annual Review of Virology 2 (2015): 67–93, 10.1146/annurev-virology-031413-085410. 26958907

[9] C. Yin , A. O. O'Reilly , S.‐N. Liu , et al., “Dual Mutations in the Whitefly Nicotinic Acetylcholine Receptor β1 Subunit Confer Target‐Site Resistance to Multiple Neonicotinoid Insecticides,” PLOS Genetics 20 (2024): e1011163, 10.1371/journal.pgen.1011163. 38377137 PMC10906874

[10] J. Yang , B. Fu , P. Gong , et al., “ *CYP*6CX2 and *CYP*6CX3 Mediate Thiamethoxam Resistance in Field Whitefly, *Bemisia tabaci* (Hemiptera:Aleyrodidae),” Journal of Economic Entomology 116 (2023): 1342–1351, 10.1093/jee/toad089. 37208311

[11] V. H. Maldonado Machado da Cruz , T. Rutz da Silva , P. Gimenez Cremonez , A. L. Biscaia Ribeiro da Silva , J. Vergilio Visentainer , and C. Rodrigues , “Tomato Secondary Metabolites as Natural Regulators of *Bemisia tabaci* Behavior and Performance: Current Applicability and Prospects,” Frontiers in Plant Science 16 (2026): 1704832, 10.3389/fpls.2025.1704832. 41626329 PMC12851975

[12] N. P. Keller , G. Turner , and J. W. Bennett , “Fungal Secondary Metabolism — From Biochemistry to Genomics,” Nature Reviews Microbiology 3 (2005): 937–947, 10.1038/nrmicro1286. 16322742

[13] W. Tang , L. Hu , and C. Pan , “Bioactive Compounds From Cyanobacteria: A Mini Review,” Current Topics in Medicinal Chemistry 26, no. 20 (2026): 2091–2101, 10.2174/0115680266436607260106072004. 41879462

[14] S.‐Y. Yang , T.‐T. Ying , T.‐H. Zhou , et al., “The Myxobacterial Genus Archangium: A Prolific and Underexploited Source of Bioactive Secondary Metabolites,” Journal of Medicinal Chemistry 68 (2025): 2183–2197, 10.1021/acs.jmedchem.4c02203. 39895639

[15] R. V. Bhadani , H. P. Gajera , D. G. Hirpara , H. J. Kachhadiya , and R. A. Dave , “Metabolomics of Extracellular Compounds and Parasitic Enzymes of Beauveria Bassiana Associated With Biological Control of Whiteflies (*Bemisia tabaci*),” Pesticide Biochemistry and Physiology 176 (2021): 104877, 10.1016/j.pestbp.2021.104877. 34119221

[16] E. Patridge , P. Gareiss , M. S. Kinch , and D. Hoyer , “An Analysis of FDA‐Approved Drugs: Natural Products and Their Derivatives,” Drug Discovery Today 21 (2016): 204–207, 10.1016/j.drudis.2015.01.009. 25617672

[17] G. Parveen , W. A. Ansari , N. Kumar , and D. K. Jaiswal , “Harnessing Secondary Metabolites of Endophytic Microbes: A Next‐Generation Biopesticide for Crop Disease Management,” Frontiers in Microbiology 16 (2026): 1705702, 10.3389/fmicb.2025.1705702. 41657995 PMC12875322

[18] X. Lyu , J. Lee , and W. N. Chen , “Potential Natural Food Preservatives and Their Sustainable Production in Yeast: Terpenoids and Polyphenols,” Journal of Agricultural and Food Chemistry 67 (2019): 4397–4417, 10.1021/acs.jafc.8b07141. 30844263

[19] A. Moeini , P. Pedram , E. Fattahi , P. Cerruti , and G. Santagata , “Edible Polymers and Secondary Bioactive Compounds for Food Packaging Applications: Antimicrobial, Mechanical, and Gas Barrier Properties,” Polymers 14 (2022): 2395, 10.3390/polym14122395. 35745971 PMC9229000

[20] J. Feng , Y. Yu , S. Huang , N. Zhu , A. Mojiri , and D. Ge , “Tannic Acid as a Green Chemical for the Removal of Various Heavy Metals: A Critical Review of Recent Developments,” Journal of Environmental Management 375 (2025): 124390, 10.1016/j.jenvman.2025.124390. 39908615

[21] N. Wang , K. Zhu , Y. Bi , F. Liu , and Z. Yu , “Anti‐Aphid Polyketides From *Streptomyces* sp. SA61,” Journal of Natural Products 86 (2023): 791–796, 10.1021/acs.jnatprod.2c00961. 36988345

[22] F. Liu , N. Wang , Y. Wang , and Z. Yu , “The Insecticidal Activity of Secondary Metabolites Produced by *Streptomyces* sp. SA61 Against *Trialeurodes Vaporariorum* (Hemiptera: Aleyrodidae),” Microorganisms 12 (2024): 2031, 10.3390/microorganisms12102031. 39458340 PMC11509760

[23] Y. Kishimura , A. Kawashima , T. Kagamizono , et al., “PI‐200 and PI‐201, New Platelet Aggregation Inhibitors Produced by *Streptomyces* sp. A7498. Taxonomy, Fermentation, Isolation, Physico‐Chemical Properties, Structure Determination and Biological Properties,” Journal of Antibiotics 45 (1992): 892–898, 10.7164/antibiotics.45.892. 1500356

[24] C. W. Cao , J. Zhang , X. W. Gao , et al., “Overexpression of Carboxylesterase Gene Associated With Organophosphorous Insecticide Resistance in Cotton Aphids, Aphis Gossypii (Glover),” Pesticide Biochemistry and Physiology 90 (2008): 175–180, 10.1016/j.pestbp.2007.11.004.

[25] T. S. Bellows , T. M. Perring , R. J. Gill , and D. H. Headrick , “Description of a Species of Bemisia (Homoptera: Aleyrodidae),” Annals of the Entomological Society of America 87, no. 2 (1994): 195–206, 10.1093/aesa/87.2.195.

