# Supplementary material for: Two New Polyketides From Streptomyces sp. SA61: Isolation, Structure Elucidation, and Insecticidal Activity Against Bemisia tabaci
Source: Chem Biodivers. 2026 Sep 15;23(9):e71706. doi: 10.1002/cbdv.71706 (PMC13576077; doi:10.1002/cbdv.71706)
Supplement: Supplementary file 1 [file CBDV-23-e71706-s001.docx]

**Two New Polyketides from *Streptomyces* sp. SA61: Isolation, Structure Elucidation, and Insecticidal Activity against *Bemisia tabaci***

Pengfei Cui^a^, Jiajun Chen^a^, Zhaoyu Zhu^a^, Fei Liu^a^, Zhiguo Yu^*, a^

（a, College of Plant Protection, Shenyang Agricultural University, Shenyang 110866, China）

**Correspondence**：Zhiguo Yu（[zyu@syau.edu.cn](mailto:zyu@syau.edu.cn)）

**Keywords**：*Bemisia tabaci*, insecticidal activity, *Streptomyces*, structural identification, secondary metabolites

**ABSTRACT**：

To discover novel green bio-based insecticidal lead compounds, six polyketides, including two new compounds, strekingmycin I (**5**) and strekingmycin J (**6**), together with four known analogues, were isolated and identified from the fermentation products of earthworm gut-derived *Streptomyces* sp. SA61. Their structures were established by comprehensive spectroscopic analysis, including NMR, HR-ESI-MS, and single-crystal ***X***-ray diffraction. Notably, the crystallographic data of strekingmycin G (**3**) were obtained for the first time, which unambiguously confirmed its absolute configuration. In insecticidal activity assays against the 3rd-instar nymphs of *Bemisia tabaci*, all six compounds exhibited varying degrees of toxicity. Among them, strekingmycin A (**1**) displayed the most potent activity, with an LC_50_ value of 5.58 μg/mL, which was within the same concentration range as that of the positive control spirotetramat, albeit less active than the commercial standard. This study enriches the structural diversity of strekingmycin-type polyketides by reporting two new derivatives and providing the first crystallographic evidence for compound **3**. Furthermore, it demonstrates for the first time the insecticidal potential of the strekingmycin family against *B. tabaci*, offering a scientific basis for further research on the management of piercing-sucking pests.

**Content**

**[Figure S1](#_Toc225793852)** ^[1](#_Toc225793852)^[H NMR Spectrum of Compound 1 (CDCl](#_Toc225793852)_[3](#_Toc225793852)_[)	1](#_Toc225793852)

**[Figure S2](#_Toc225793853)** ^[13](#_Toc225793853)^[C NMR Spectrum of Compound](#_Toc225793853) **[1](#_Toc225793853)** [(CDCl](#_Toc225793853)_[3](#_Toc225793853)_[)	1](#_Toc225793853)

**[Figure S3](#_Toc225793854)** ^[1](#_Toc225793854)^[H NMR Spectrum of Compound](#_Toc225793854) **[2](#_Toc225793854)** [(CDCl](#_Toc225793854)_[3](#_Toc225793854)_[)	2](#_Toc225793854)

**[Figure S4](#_Toc225793855)** ^[13](#_Toc225793855)^[C NMR Spectrum of Compound](#_Toc225793855) **[2](#_Toc225793855)** [(CDCl](#_Toc225793855)_[3](#_Toc225793855)_[)	2](#_Toc225793855)

**[Figure S5](#_Toc225793856)** ^[1](#_Toc225793856)^[H NMR Spectrum of Compound](#_Toc225793856) **[3](#_Toc225793856)** [(CDCl](#_Toc225793856)_[3](#_Toc225793856)_[)	3](#_Toc225793856)

**[Figure S6](#_Toc225793857)** ^[13](#_Toc225793857)^[C NMR Spectrum of Compound](#_Toc225793857) **[3](#_Toc225793857)** [(CDCl](#_Toc225793857)_[3](#_Toc225793857)_[)	3](#_Toc225793857)

**[Figure S7](#_Toc225793858)** ^[1](#_Toc225793858)^[H NMR Spectrum of Compound](#_Toc225793858) **[4](#_Toc225793858)** [(CDCl](#_Toc225793858)_[3](#_Toc225793858)_[)	4](#_Toc225793858)

**[Figure S8](#_Toc225793859)** ^[13](#_Toc225793859)^[C NMR Spectrum of Compound](#_Toc225793859) **[4](#_Toc225793859)** [(CDCl](#_Toc225793859)_[3](#_Toc225793859)_[)	4](#_Toc225793859)

**[Figure S9](#_Toc225793860)** ^[1](#_Toc225793860)^[H NMR Spectrum of Compound](#_Toc225793860) **[5](#_Toc225793860)** [(CDCl](#_Toc225793860)_[3](#_Toc225793860)_[)	5](#_Toc225793860)

**[Figure S10](#_Toc225793861)** ^[13](#_Toc225793861)^[C NMR Spectrum of Compound](#_Toc225793861) **[5](#_Toc225793861)** [(CDCl](#_Toc225793861)_[3](#_Toc225793861)_[)	5](#_Toc225793861)

**[Figure S11](#_Toc225793862)** [The](#_Toc225793862) ^[1](#_Toc225793862)^[H-](#_Toc225793862)^[1](#_Toc225793862)^[H COSY Spectrum of Compound](#_Toc225793862) **[5](#_Toc225793862)** [(CDCl](#_Toc225793862)_[3](#_Toc225793862)_[)	6](#_Toc225793862)

**[Figure S12](#_Toc225793863)** [The HSQC Spectrum of Compound](#_Toc225793863) **[5](#_Toc225793863)** [(CDCl](#_Toc225793863)_[3](#_Toc225793863)_[)	6](#_Toc225793863)

**[Figure S13](#_Toc225793864)** [The HMBC Spectrum of Compound](#_Toc225793864) **[5](#_Toc225793864)** [(CDCl](#_Toc225793864)_[3](#_Toc225793864)_[)	7](#_Toc225793864)

**[Figure S14](#_Toc225793865)** [The NOESY Spectrum of Compound](#_Toc225793865) **[5](#_Toc225793865)** [(CDCl](#_Toc225793865)_[3](#_Toc225793865)_[)	7](#_Toc225793865)

**[Figure S15](#_Toc225793866)** [The HRESIMS Spectrum of Compound](#_Toc225793866) **[5](#_Toc225793866)**	[8](#_Toc225793866)

**[Figure S16](#_Toc225793867)** ^[1](#_Toc225793867)^[H NMR Spectrum of Compound](#_Toc225793867) **[6](#_Toc225793867)** [(CDCl](#_Toc225793867)_[3](#_Toc225793867)_[)	8](#_Toc225793867)

**[Figure S17](#_Toc225793868)** ^[13](#_Toc225793868)^[C NMR Spectrum of Compound](#_Toc225793868) **[6](#_Toc225793868)** [(CDCl](#_Toc225793868)_[3](#_Toc225793868)_[)	9](#_Toc225793868)

**[Figure S18](#_Toc225793869)** [The](#_Toc225793869) ^[1](#_Toc225793869)^[H-](#_Toc225793869)^[1](#_Toc225793869)^[H COSY Spectrum of Compound](#_Toc225793869) **[6](#_Toc225793869)** [(CDCl](#_Toc225793869)_[3](#_Toc225793869)_[)	9](#_Toc225793869)

**[Figure S19](#_Toc225793870)** [The HSQC Spectrum of Compound](#_Toc225793870) **[6](#_Toc225793870)** [(CDCl](#_Toc225793870)_[3](#_Toc225793870)_[)	10](#_Toc225793870)

**[Figure S20](#_Toc225793871)** [The HMBC Spectrum of Compound](#_Toc225793871) **[6](#_Toc225793871)** [(CDCl](#_Toc225793871)_[3](#_Toc225793871)_[)	10](#_Toc225793871)

**[Figure S21](#_Toc225793872)** [The NOESY Spectrum of Compound](#_Toc225793872) **[6](#_Toc225793872)** [(CDCl](#_Toc225793872)_[3](#_Toc225793872)_[)	11](#_Toc225793872)

**[Figure S22](#_Toc225793873)** [The HRESIMS Spectrum of Compound](#_Toc225793873) **[6](#_Toc225793873)**	[11](#_Toc225793873)

**Figure S1** ^1^H NMR Spectrum of Compound **1** (CDCl_3_)

**Figure S2** ^13^C NMR Spectrum of Compound **1** (CDCl_3_)

**Figure S3** ^1^H NMR Spectrum of Compound **2** (CDCl_3_)

**Figure S4** ^13^C NMR Spectrum of Compound **2** (CDCl_3_)

**Figure S5** ^1^H NMR Spectrum of Compound **3** (CDCl_3_)

**Figure S6** ^13^C NMR Spectrum of Compound **3** (CDCl_3_)

**Figure S7** ^1^H NMR Spectrum of Compound **4** (CDCl_3_)

**Figure S8** ^13^C NMR Spectrum of Compound **4** (CDCl_3_)

**Figure S9** ^1^H NMR Spectrum of Compound **5** (CDCl_3_)

**Figure S10** ^13^C NMR Spectrum of Compound **5** (CDCl_3_)

**Figure S11** The ^1^H-^1^H COSY Spectrum of Compound **5** (CDCl_3_)

**Figure S12** The HSQC Spectrum of Compound **5** (CDCl_3_)

**Figure S13** The HMBC Spectrum of Compound **5** (CDCl_3_)

**Figure S14** The NOESY Spectrum of Compound **5** (CDCl_3_)

**Figure S15** The HRESIMS Spectrum of Compound **5**

**Figure S16** ^1^H NMR Spectrum of Compound **6** (CDCl_3_)

**Figure S17** ^13^C NMR Spectrum of Compound **6** (CDCl_3_)

**Figure S18** The ^1^H-^1^H COSY Spectrum of Compound **6** (CDCl_3_)

**Figure S19** The HSQC Spectrum of Compound **6** (CDCl_3_)

**Figure S20** The HMBC Spectrum of Compound **6** (CDCl_3_)

**Figure S21** The NOESY Spectrum of Compound **6** (CDCl_3_)

**Figure S22** The HRESIMS Spectrum of Compound **6**
